# Mismatch oligonucleotides in human and yeast: guidelines for probe design on tiling microarrays

**DOI:** 10.1186/1471-2164-9-635

**Published:** 2008-12-31

**Authors:** Michael Seringhaus, Joel Rozowsky, Thomas Royce, Ugrappa Nagalakshmi, Justin Jee, Michael Snyder, Mark Gerstein

**Affiliations:** 1 Department of Molecular Biophysics and Biochemistry, Yale University, New Haven, CT 06520, USA; 2 Program in Computational Biology and Bioinformatics, Yale University, New Haven, CT 06520, USA; 3 Department of Molecular, Cellular and Developmental Biology, Yale University, New Haven, CT 06520, USA; 4 Department of Computer Science, Yale University, New Haven, CT 06520, USA

## Abstract

**Background:**

Mismatched oligonucleotides are widely used on microarrays to differentiate specific from nonspecific hybridization. While many experiments rely on such oligos, the hybridization behavior of various degrees of mismatch (MM) structure has not been extensively studied. Here, we present the results of two large-scale microarray experiments on *S. cerevisiae *and *H. sapiens *genomic DNA, to explore MM oligonucleotide behavior with real sample mixtures under tiling-array conditions.

**Results:**

We examined all possible nucleotide substitutions at the central position of 36-nucleotide probes, and found that nonspecific binding by MM oligos depends upon the individual nucleotide substitutions they incorporate: C→A, C→G and T→A (yielding purine-purine mispairs) are most disruptive, whereas A→X were least disruptive. We also quantify a marked GC skew effect: substitutions raising probe GC content exhibit higher intensity (and vice versa). This skew is small in highly-expressed regions (± 0.5% of total intensity range) and large (± 2% or more) elsewhere. Multiple mismatches per oligo are largely additive in effect: each MM added in a distributed fashion causes an additional 21% intensity drop relative to PM, three-fold more disruptive than adding adjacent mispairs (7% drop per MM).

**Conclusion:**

We investigate several parameters for oligonucleotide design, including the effects of each central nucleotide substitution on array signal intensity and of multiple MM per oligo. To avoid GC skew, individual substitutions should not alter probe GC content. RNA sample mixture complexity may increase the amount of nonspecific hybridization, magnify GC skew and boost the intensity of MM oligos at all levels.

## Background

Oligonucleotide tiling arrays are a popular tool for detecting transcriptionally active regions on a genomic scale. They comprise short oligomeric probes (generally 25–70 bp) immobilized on a slide surface; a typical custom-built tiling array today contains about 400,000 features. Tiling arrays are distinct from traditional microarrays, which are most often used to measure differential gene expression in multiple biological conditions. As such, different techniques must be employed in their analysis [1].

The principle behind microarray analysis is similar to that of traditional hybridization using nitrocellulose membranes [2]: When fluorescently-labeled sample (target) is applied to the array-bound features (probes), complementary regions of probe and target DNA will anneal to form a stable duplex. Thus, any probe whose complementary target is present in the sample mixture should bind fluorescent label.

However, observed fluorescent intensity (signal) can derive not only from such gene-specific binding, but also from non-specific binding. This occurs when target DNA anneals in a nonspecific manner to the probe. Non-specific binding is dependent upon probe sequence, but independent of the amount of its true target in the sample; thus, it is unrelated to the transcriptional activity or expression level of the gene it is designed to assay.

Controlling for such background hybridization is an ongoing concern in microarray studies, and particularly important for tiling arrays where the absolute intensity (as opposed to relative intensity of two samples) is sought and where less discretion is allowed in probe selection. Traditional microarrays typically include only a handful of probes per gene, which can be carefully chosen to optimize binding and target discrimination. In tiling arrays however, such probe choice is impossible, and as such mismatch (MM) probes become an important tool to enhance probe performance.

MM are often included on microarrays to differentiate specific from nonspecific hybridization. The rationale for their inclusion relies on three assumptions: first, that nonspecific binding affects perfect match (PM) and MM probes equally; second, that the mismatch reduces the affinity of gene-specific binding to the MM; and third, that fluorescence signal per bound transcript is identical for PM and MM [3]. In Affymetrix GeneChips^® ^for example, the central base of a 25 mer probe is replaced by its complement; subtracting MM signal from PM intensity (PM-MM) is meant to yield true probe signal corrected for non-specific background. However, MM signal is often greater than PM signal [4] and either ignored entirely [5,6] or simply used to exclude outliers.

All nucleotide substitutions do not have an equal impact on hybridization efficiency. Previous work has explored these differences in solution [7] and *in silico *[8] using solution-phase algorithms, but solution-phase kinetics do not translate well to solid phase hybridization [9].

While many experiments include probes with single MMs, and much previous work has focused on trying to model hybridization on microarrays [3,9], the hybridization behavior of various degrees of MM structure on arrays remains poorly understood. More elaborate mismatch strategies for oligonucleotide microarrays have been investigated in the context of resequencing and mutational analysis [10]. Moreover, experiments examining MM behavior on arrays often rely on spike-in [11] or custom synthesized sample mixtures [7,12], which limit non-specific hybridization and differ from the complex mixtures normally present in array experiments.

In array design, a great deal of work has focused on selecting the best probes from genomic DNA. Several software programs exist to design oligos for microarrays: CommOligo [13], ArrayOligoSelector , OligoArray [14], OligoArray 2.0 [15], and OligoPicker [16]. Factors considered include maximizing probe uniqueness in the genome to minimize cross-hybridization, and altering probe length to conform to a needed range of melting temperature. Little thought is given to selecting nucleic acid substitutions for MM probes; typically, the PM middle base's complement is chosen, without consideration for the potential differences in signal that can arise from these choices.

The behavior of multiple MM per probe has not been deeply explored. In 2003, DiRisi and coworkers examined the effects of multiple MM on 70 mer probes arranged according to two schemes: internal (distributed at random along the length of the probe), and anchored (contiguous at either end) [17]. While the relative intensity in the anchored MM set decreased gradually as more MM were added, the internal MM set disrupted hybridization much more efficiently and fell off too rapidly to extract the cost per MM.

Here, we present the results of two large-scale microarray experiments to explore MM oligonucleotide behavior with real sample mixtures under tiling-array conditions. We designed two microarrays to assess the performance of MM oligonucleotides, one design using the Baker's yeast *Saccharomyces cerevisiae *(probed with total RNA), and the other design using human genomic material (probed with placental RNA). MM probes were selected from a set of fourteen designs. These include three SingleCenter designs (center position 18 of each 36-mer changed to all three possible substitute nucleotides), five centered designs (3, 5, 7, 9, and 11 MMs arranged about the center position in a contiguous group), five staggered designs (2, 3, 4, 5 and 7 MMs distributed evenly throughout the 36-mer) and one deletion of the central position. Experiments were carried out in triplicate, and the data has been deposited in the GEO database (GEO accession GSE13175).

The yeast array includes a 10 kb region surrounding the highly-expressed ACT1 gene, tiled double-stranded with 36-mer probes at 1 bp spacing. For each PM oligo derived from this gene's sequence, all fourteen MM oligos designs were included. The yeast array also includes six additional genes, tiled single-stranded with 36-mer probes at 1 bp spacing, with a smaller set of four MM oligos per PM. The human array includes three RefSeq genes (HBG2, TIMP3, SYN3) with flanking regions, tiled double-stranded with 36-mer probes at 1 bp spacing (similar to ACT1, above). Thirteen MM oligos were included for each human PM probe (the deletion MM was not included in the human design).

We analyze the effect of each MM substitution under real conditions, and examine the impact of including multiple MM in a single probe. Though there do exist some caveats – namely, that the conclusions presented here may not apply exactly to different array types, oligonucleotide lengths, labeling methods and so on – these results will be useful in computing nonspecific hybridization of individual probes, and as a general guideline for designing MM probes for tiling arrays: a new and exciting application of array technology, where single MM probes no longer suffice.

## Results

Genomic regions from seven *S. cerevisiae *genes and three human genes were tiled with perfect match (PM) and mismatch (MM) oligos, the arrays were probed and the resulting intensities normalized as outlined in the Methods section.

**Figure 1:**
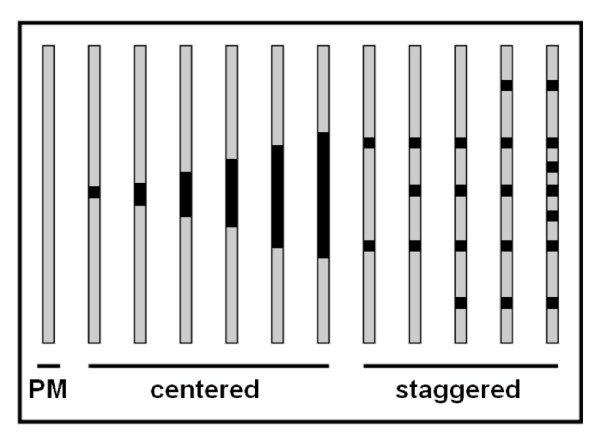
**Mismatch schematic**. Schematic of mismatch oligo design. Black represents mismatch positions. SingleCenter is the leftmost centered design, and comprises three distinct oligo designs: one for each central base substitution.

Four distinct classes of MM oligo were employed: *SingleCenter*, wherein the nucleotide at the center position (18) is changed; *centered*, wherein mismatch bases are added incrementally from the center of the oligo; *staggered*, wherein mismatch bases are distributed throughout the length of the oligo; and *deletion*, wherein the center position (18) is removed, and the oligo is elongated by adding the next base in genomic sequence. The location of mismatch bases in these designs is given in Table 1, and schematized in Figure 1. The aforementioned genes were tiled with various sets of these MM designs.

**Table 1:** Mismatch designs

**DESIGN**	**# MMs**	**MM BASE LOCATIONS**
PM	0	--

Centered		

c1	1	18
c3	3	17–19
c5	5	16–20
c7	7	15–21
c9	9	14–22
c11	11	13–23

Staggered		

s2	2	12, 24
s3	3	12, 18, 24
s4	4	6, 12, 18, 14
s5	5	6, 12, 18, 24, 30
s7	7	6, 12, 15, 18, 21, 24, 30

Design of mismatch oligo classes: SingleCenter (c1), centered (c2-c11) and staggered (s2-s7).

**Figure 2:**
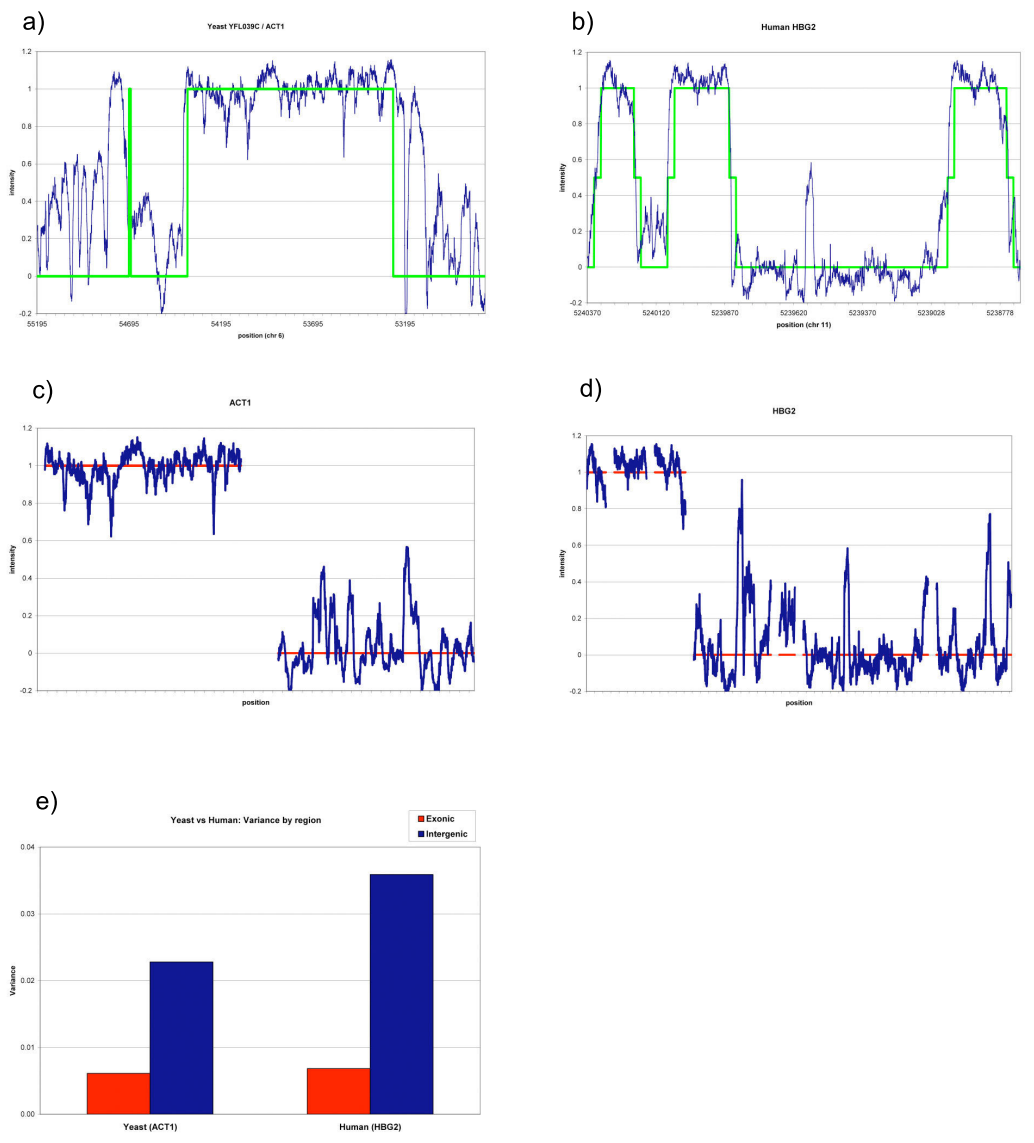
**Gene regions & mixture complexity**. (a, b) Signal plots (blue) for yeast ACT1 (a) and human HBG2 (b) genes, showing annotated coding regions (green). (Flanking regions not shown.) (c, d) Intensity normalization: Representative coding (left) and non-coding (right) regions selected from genes (including flanking regions) and used for normalization from ACT1 (c) and HBG2 (d). Median intensity in coding regions was set to 1, and median intensity in non-coding regions was set to 0 (red line). All slide intensities were normalized to these values. (e) Mixture complexity: Normalized intensity variance for ACT1 and HBG2. Increased mixture complexity in human corresponds to larger signal variance, particularly in low expressed regions.

Our normalization scale equates median log intensity of high-expression regions to 1.0, and median log intensity of non-coding regions (background) to 0.0 (Figure 2a–d); thus, an oligo with intensity equal to that of a highly-expressed region will be assigned a score of ~1, while an intensity near baseline will score ~0. MM oligos were evaluated by taking the difference in normalized intensities (MM-PM). This value is expressed as a percentage of the total intensity range. (Thus, if the normalized PM probe intensity is 0.8, and the normalized MM intensity is 0.6, the MM-PM difference will be -0.2 on our normalized scale, which equates to 20% of the total normalized intensity range.) Original data is available from GEO (accession GSE13175).

### Single Center MMs

**Figure 3:**
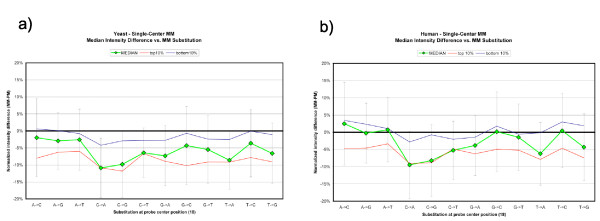
**Effect on probe intensity of all possible single-base substitutions**. Median intensity difference vs. MM substitution for all assayed regions in yeast (a) and human (b). Green curve is median intensity difference (MM-PM) for all instances of substitutions listed on the x-axis. Error bars represent ± σ for this curve. Blue curve: median intensity difference for high-intensity spots only (top 10% of spots by PM intensity). Red curve: median intensity difference for low-intensity spots (bottom 10% of spots by PM intensity).

Behavior of each possible nucleotide substitution was assessed with SingleCenter MM oligos. Figure 3 shows the cost per individual replacement, assessed over all regions tiled (seven yeast genes and three human genes).

In both species, C→A was the most disruptive change (-11% yeast, -9% human), followed by C→G (-10% yeast, -8% human) and T→A (-9% yeast, -6% human); G→A, T→G and C→T were the next most disruptive changes. The least disruptive changes were those beginning with adenine, A→C (-2% yeast, +3% human), A→T (-3% yeast, +1% human), and A→G (-3% yeast, no change in human), followed by T→C, G→C, and G→T. In human, the two least disruptive MMs actually increased median spot intensity relative to PM. It should be emphasized that a wide range of values was found in each case (note standard deviation bars in Figure 3). The aforementioned values are the median intensity changes.

### GC skew

**Figure 4:**
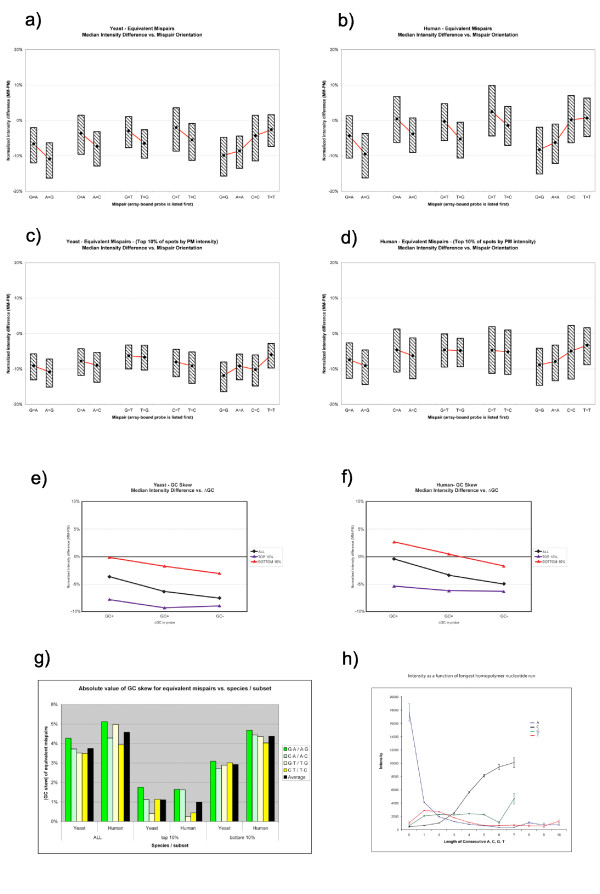
**GC skew in equivalent substitutions**. *(a-d) *Median intensity difference vs. mispair orientation for equivalent mispairs in yeast (a, c) and human (b, d). For each mispair, the base listed first is contained in the array-bound oligo, while the second is its presumed counterpart in solution. Mispairs are plotted in the order shown in Table 2. Boxes represent inter-quartile range (i.e. middle 50% of intensity values for each mispair lie between upper and lower bounds). Figure gives mispair skew for all assayed regions (a, b), and for high-intensity (c, d) spots only. *(e, f) *Median intensity difference vs. GC effect in yeast (e) and human (f): plots those substitutions which increase GC content of oligo (GC+, left side of graph), those with no effect on GC content (GC =, middle) and those decreasing GC content of oligo (GC-, right). *(g) *Absolute value of the GC skew between equivalent mispairs presented in (a-f), with average skew by species and subset. *(h) *Intensity as a function of the longest length of a homopolymer nucleotide run contained within the sequence of an oligonucleotide probe. A (blue), C (black), G (green) and T (red) are displayed. Human PM data is shown here.

SingleCenter nucleotide substitutions were grouped into equivalent mispairs as shown in Table 2. The median intensity difference (MM-PM) vs. mispair orientation is given in Figure 4b–e. A consistent skew is seen: nucleotide substitutions that increase the GC content of the 36 mer probe have a higher median intensity than those that decrease it or leave it unchanged. This trend is seen across all tiled regions (Figure 4a, b), in the highest intensity spots (Figure 4c, d) and the lowest intensity spots as well (data not shown).

**Table 2:** Table of GC skew and equivalent substitutions

Substitution	T→G	C→A	T→C	G→A	A→G	C→T	A→C	G→T	C→G	T→A	G→C	A→T
Mispair	G-A	A-G	C-A	A-C	G-T	T-G	C-T	T-C	G-G	A-A	C-C	T-T
GC Effect	+	-	+	-	+	-	+	-	=	=	=	=

All possible single nucleotide substitutions, grouped into equivalent mispairs (a): base substitution (top, shown as original→substitution) leads to mispair (middle row) with resulting effect on GC content of oligo (bottom, + for increase in oligo GC content, - for decrease).

The twelve individual SingleCenter nucleotide substitutions were then regrouped into three classes according to their effect on the GC content of the oligo. Overall, the median intensity difference of a single substitution at position 18 can vary between -4% and -8% in yeast depending on its effect on GC content (Figure 4e). In low expression regions, this spread remains pronounced (red line; between 0% and -3%) whereas in highly expressed regions, the spread is narrower (blue line; between -8% and -9%). In human (Figure 4f) the same effect is seen. Overall, the median intensity difference of a single center substitution varies between 0% and -5% depending on GC effect; in low expression regions, this range shifts upwards (from +3% to -2%) and in highly expressed regions the skew is lessened (-5% to -6%).

### Non-specific binding due to homopolymer C nucleotide runs

We have found that oligonucleotides that contain consecutive runs of C nucleotides longer than or equal to four show abnormally high fluorescent intensity for the case of the human PM data. In Figure 4h we plot the average intensity of probes containing a homopolymer run of nucleotides (either A, C, G or T) of a given length. We observe that the hybridization intensities of oligonucleotides containing a run of four or more C's are significantly higher than the average array signal intensity. This effect is not simply due to these oligonucleotide probes containing large amounts of C nucleotides: we have observed that probes specifically with runs of four or more C have higher intensities than oligonucleotides with the same C nucleotide content but lacking a contiguous run of C. We also observe that oligonucleotide probes with no A show enriched signal intensities.

This effect does not appear to be caused by direct binding of oligonucleotides to reverse complements by Watson-Crick base pairing, since this effect is not present for consecutive runs of G's. We hypothesize that runs of C's cause oligonucleotide probes to exhibit non-specific cross-hybridization to targets other than their reverse complement. Potentially, this binding might be more exotic than regular Watson-Crick base pairing. This effect has been observed for other sets of human tiling array data, however this effect is not present for the yeast PM data. One possible explanation for the enhanced signals from oligonucleotides probes with poly-C runs is offered by Nelson et al. in 2007 [18], in a study which identified an artifact caused by the T7 primer used by the in vitro transcription system that caused certain oligonucleotide probes to yield increased signal. However, this still does not explain the differences in poly-C intensity observed between human and yeast arrays.

When designing tiling microarrays, care should be taken to avoid oligonucleotide sequences containing runs of consecutive C, since these may not probe only intended targets and may instead yield ambiguous signals.

### Deletion MMs

Deletion MMs were found to have an effect similar to SingleCenter MMs. Full results are available in supplementary material online.

### Multiple MMs

**Figure 5:**
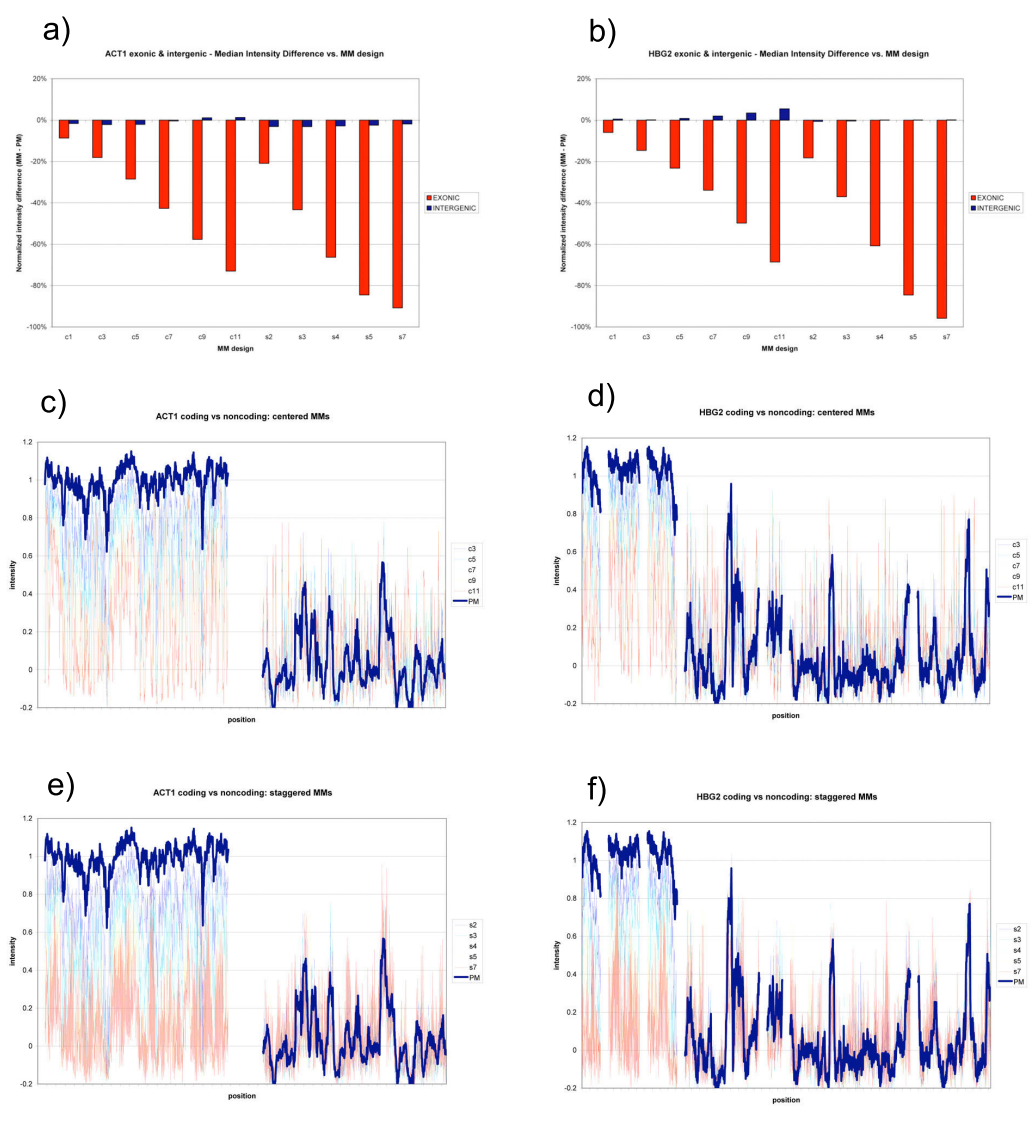
**MM vs PM**. (a, b) Median intensity difference (MM-PM) vs. mismatch design for yeast ACT1 (a) and human HBG2 (b) genes. Mismatch oligos were divided into two groups: those taken from exonic regions are shown in red; intergenic regions, shown in blue. (c, d) Centered MM intensities superimposed on PM for coding (left) and non-coding (right) regions. (e, f) Staggered MM intensities superimposed on PM for coding (left) and non-coding (right) regions.

For multiple MM studies, we focused on two individual highly expressed genes, yeast ACT1 and human HBG2. These were partitioned into exonic and intergenic regions by annotation reference (this partitioning was supported by observed array intensities). The median intensity difference for each MM design is given in Figure (5a, b).

In exonic regions (red), additional mismatches cause a downward trend in intensity relative to the PM. In intergenic regions, mismatches show no distinct trend, but instead fluctuate about the PM intensity.

Figure 5(c–f) shows the signal plots for centered (c, d) and staggered (e, f) MMs in yeast and human, superimposed on a PM trace. The regions shown are the same coding and non-coding excerpts given in Figure 2.

### Cost per additional MM

**Figure 6:**
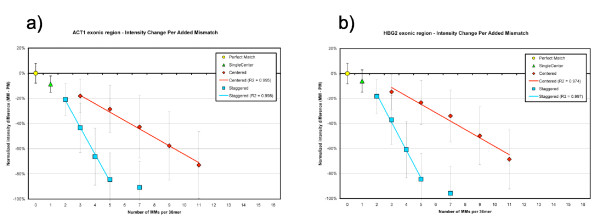
**Intensity change per mismatch**. Intensity change per added mismatch in exonic region of yeast ACT1 (a) and human HBG2 (b). Error bars represent ± σ.

Figure 6 plots the median intensity difference for each mismatch design relative to PM for exonic DNA in yeast ACT1 (a) and human HBG2 (b). We computed the cost per *n *MMs for each scheme by fitting each to a linear equation, expressed in terms of normalized intensity difference *i *(MM-PM). In ACT1, the five multiple MM designs in the centered scheme (c3, c5, c7, c9, c11) fit to *i *= -0.0669*n *+ 0.0253 with R^2 ^= 0.995, whereas the four staggered MM designs (s2, s3, s4, s5) fit to *i *= -0.2138*n *+ 0.2108 with R^2 ^= 0.998. In HBG2, the centered designs fit to *i *= -0.0672*n *+ 0.0902 (R^2 ^= 0.974) and the four staggered designs fit to *i *= -0.2227*n *+ 0.2781 with (R^2 ^= 0.997). In both species, the fifth staggered design (s7) was excluded from the fit, since the observed linear behavior plateaus near baseline intensity.

Extracting the slopes of these curves, we determine that each additional mismatch in the centered scheme costs ~7% of PM intensity in both yeast and human, whereas each mismatch added in a staggered scheme is approximately three-fold more disruptive (cost is ~21% per MM in yeast and human).

### Using MM to discern coding regions

To assess the performance of the various MM classes at discerning transcribed sequence, a set of pair-wise t-tests were performed. A 0.5 kb region (comprising 512 distinct probes) was selected from within a known coding region, and contrasted with a non-coding region of equal size. A t-test was performed for each MM with the PM. This was done for two genes, ACT1 (yeast) and HBG2 (human).

In known coding regions, all MMs differed significantly from the PM (p < 1 × 10^-100 ^in all cases). The s7 design (7 mismatched bases staggered throughout the oligo) was the most different from PM (off scale, p = 0 in both yeast and human). In known non-coding regions, MMs displayed a range of performance: some were significantly different from PM (in particular the c11 design, p = 2 × 10^-6 ^in yeast, p = 3 × 10^-5 ^in human), whereas the s7 design was indistinguishable from PM in both cases (p = 0.21 in yeast, p = 0.08 in human).

Because the windows for scoring transcribed fragments are often short (5–10 probes), the above assay was repeated with 10 probes per window. The same pattern was observed: in a window containing known coding sequence, all MM classes could be distinguished from PM (p <= 1 × 10^-4 ^in all cases), with the s7 design displaying the smallest p-value (p = 2 × 10^-9 ^in yeast, p = 8 × 10^-11 ^in human). In non-coding windows, most MM classes were indistinguishable from PM, and again the s7 design had the highest p-values (p = 0.72 in yeast, p = 0.37 in human).

## Discussion

Oligonucleotide probes containing single mismatch (MM) positions are frequently included in microarrays to control for nonspecific hybridization. Relative to the perfect match (PM), the mismatch oligo should exhibit much less gene-specific binding, but a similar amount of nonspecific binding; thus, subtracting the MM signal should yield a true PM corrected for nonspecific binding. In practice, selecting the number, type and placement of MM in a given probe is challenging, particularly in the case of tiling arrays which permit far less flexibility in probe selection than traditional expression arrays. We have conducted microarray experiments in two species to demonstrate the behavior of various types of MM design under normal experimental conditions. These results represent a formal examination of previously unstructured and largely anecdotal microarray knowledge, and a resource useful for array design.

### Single MMs at the central position

We demonstrate here that the amount of nonspecific binding exhibited by MM oligos is dependent upon the individual nucleotide substitutions they incorporate, and therefore not necessarily equivalent to that experienced by the PM probe – this finding undercuts a main assumption underlying the use of MM probes. For each of the four DNA nucleotides (A, C, G, and T) there exist three possible substitutions, and we find that these twelve possible replacements exhibit a range of effect on MM intensity. Some of these are anticipated: for instance, sterically unfavorable purine-purine mispairs such as A·G are expected to destabilize duplex DNA more than purine-pyrimidine mispairs. Our findings support this, with the three purine-purine mispairs – C→A (yielding the A·G mispair), C→G (G·G mispair) and T→A (A·A mispair) – emerging as the most disruptive single substitutions.

However, we are also able to revise and refine predictions stemming from earlier work. Using *in silico *hybridization models of single-point mismatches, Athey and coworkers (2004) [8] predicted that G→X would be the substitution most disruptive to oligonucleotide hybridization, while A→G and T→G would be the least. We report here that in practice, C→X is most disruptive, and the three A→X changes are least disruptive. Zhang and coworkers reported that although G·G is more stable than C·C in solution, the reverse is true on an array [11]. We confirm that the G→C substitution (C·C mispair) is more stable than the C→G substitution (G·G mispair), further supporting the notion that solution-phase values do not necessarily translate to solid-phase hybridization [9].

These results are worthwhile to consider when making single MM changes, particularly when the position to be substituted is C (greatest effect) or A (smallest effect).

### GC skew effect

A notable contribution to the spread of intensities from single-point mismatches is a phenomenon we term the *GC skew *effect. With the exception of homogeneous mispairs (C·C, G·G, A·A and T·T), any given non-Watson-Crick mispair can be produced via two distinct mismatch substitutions (e.g., C→A and T→G both yield the A·G mispair). We term these pairs of changes *equivalent substitutions*. In the absence of nonspecific binding, cross-hybridization, or other array effects, such equivalent substitutions should yield equivalent spot intensity: an A·G mispair, no matter which (the A or the G) was introduced as a MM, should disrupt binding consistently. Thus, by examining the intensity differences between equivalent substitutions in aggregate, we can quantify the contribution of factors other than specific binding to observed oligo intensity.

To illustrate this phenomenon, single-point nucleotide substitutions are grouped into dyads, each representing an equivalent substitution. What emerges is a consistent skew, which can be stated as follows: All else equal, any substitution that elevates probe GC content will exhibit higher intensity, whereas any substitution that lowers probe GC content will decrease intensity.

We expect the GC skew effect to be more pronounced both in low-expressed regions – where nonspecific binding is a large component of total signal intensity – and in experiments where high mixture complexity of sample RNA provides increased potential for nonspecific hybridization, such as human arrays. We indeed find that in highly-expressed regions the average GC skew is small (±~0.5%) and similar in both human and yeast; but in low-expressed regions the average skew is markedly larger in yeast (±~1.5%) and larger still (±~2.2%) in human.

GC skew likely stems from a combination the increased 'stickiness' of the triple-H-bond arrangement of G and C nucleotides, combined with mixture complexity. (Sample labeling is likely not a contributing factor to GC skew, because on tiling arrays end-labelling is frequently used.) A complex sample RNA mixture will offer ample substrate for nonspecific hybridization.

It is evident that GC skew can constitute a large component of the effect of any single mismatch. Therefore, if employing mismatches to control for nonspecific hybridization, one should take care to make substitutions that do not alter the GC content relative to the PM oligonucleotide. That is, individual bases should be substituted with their Watson-Crick complements (A→T, C→G, G→C, T→A). This is the substitution matrix employed by Affymetrix for GeneChip^® ^arrays. It should be noted that while insulated from GC bias, these substitutions do nonetheless exhibit differing effects on intensity: in yeast, the maximal intensity spread between C→G (G·G mispair) and A→T (T·T mispair) is ~7%; in human, it can reach ~9%.

Companies such as Affymetrix^® ^have for some time insulated single-MM probes from GC bias in this manner. However, the full formalism for why and how this matrix is effective has not previously been reported in the literature. We demonstrate here why this approach is valuable and should be replicated where possible when constructing multiple-MM oligos. (A skew does remain, however, in the relative intensities of MM oligos depending upon the original nucleotide at the substituted position, as stated in the previous section.)

### Multiple MMs

In addition to all possible single-point substitutions at the center position, we also assayed ten MM oligo designs with multiple mismatches arranged in two schemes: centered and staggered. We report here that on the normalized log scale used in this work, both schemes conform to a fundamentally linear decay of signal, suggesting that multiple mismatches display a largely additive effect.

Because efficient duplex hybridization requires an uninterrupted run of complementary nucleotides, we expect the staggered scheme to be most disruptive [12,17]. This is evident in the results: each additional staggered MM causes > 20% intensity drop, whereas each centered MM causes only 7% intensity drop. The staggered curve is linear (R^2 ^= 0.998 yeast, R^2 ^= 0.997 human) from 2 through 5 mismatches, and diverges at the 7 MM level. This behavior is in line with expectation: oligonucleotides with the maximum possible number of mismatches (i.e. with every position changed) would share no sequence in common with the PM and should, on average, display signal in line with slide background intensity – the signal displayed by DNA regions that are not transcribed and thus should not be present in the target mixture. Thus, the theoretical lower bound intensity for unlimited mismatches is the slide background intensity, equivalent to 0 on our normalized log scale. In reality this lower bound is reached quite early: with 7 staggered mismatches, the signal has already diverged from the linear progression. For centered MM, by extrapolating the linear fit we predict that this scheme should begin to plateau around 15 MM per 36-mer probe; after which point additional MM should no longer reliably affect hybridization signal intensity.

Although signal decay from centered mismatches is linear to a good approximation, a slight synergistic effect is seen: Centered MM progressions in both yeast and human deviate somewhat from true linearity, suggesting some cooperativity between multiple mismatches in this scheme (this effect is more pronounced in human). This agrees with an earlier study, which suggests a synergistic effect between multiple mismatches [8]. This is understandable since centered MMs are introduced adjacent to one another, creating a contiguous run of MMs whose effect may be slightly more than the sum of its parts. In contrast, staggered MMs are introduced in isolated locations, and display near-perfect linear decay, suggesting a lack of cooperativity in this scheme.

The intercepts of the fit lines for the centered MM scheme provide an interesting insight into nonspecific hybridization behavior. In yeast and human, these lines have the same slope (7% normalized intensity decrease per mismatch), but extrapolating back to *n *= 0 MMs yields an intensity higher than that of the true PM in either case. This suggests that MM oligos pick up more signal than they should due to nonspecific hybridization, and that the amount of this signal boost is consistent at each *n*. The amount of this signal boost is encapsulated in the y-intercept value: These are different in yeast (+2.5%) and human (+9.0%), suggesting a differing amount of non-specific hybridization is contributing in each case. The increased RNA sample complexity in human may account for the higher signal boost observed at every MM level.

In intergenic (noncoding) regions, staggered mismatches show no distinct progression, but instead fluctuate about the baseline intensity. This is in line with expectation: in non-transcribed regions, deviation from the PM oligo should be no more or less likely, on average, to base-pair with material in the RNA sample mixture. The centered scheme however displays a slight upward intensity trend in intergenic regions. This cannot be explained by GC skew or general nonspecific binding, and presents an interesting matter for further inquiry.

### Identifying transcribed regions: Which MM is best?

A typical goal when employing MM oligos on an array is to help distinguish transcribed from untranscribed regions. A good MM design will thus yield intensities significantly different from PM in transcribed regions, and close to or indistinguishable from PM in untranscribed areas. We assessed the behavior of the various MM classes with respect to this task, and found that while all MM classes could be distinguished from PM in short (10 oligo) or long (512 oligo) windows of known coding sequence, their performance in non-coding windows varied. The s7 design (7 mismatched bases staggered throughout the oligo) performed best in both human and yeast: its p-value was smallest (i.e., most distinguishable from PM) across known coding sequence, and largest (i.e., least distinguishable from PM) in non-coding sequence. If generally applicable, this result suggests that a design bearing multiple staggered mismatches should outperform the traditional single-center mismatch oligos in current use, with respect to discerning transcribed sequence.

This is true in aggregate, but what about taking each oligo on its own? In aggregate, MM are not needed, since instead randomized oligos could be put down to yield background signal which could then be subtracted out. This however does not preserve the (nonspecific) binding characteristics of the PM, which is the idea underlying the MM.

## Conclusion

We have conducted two large-scale microarray experiments to explore mismatch oligonucleotide behavior with real sample mixtures under tiling-array conditions. We show that the amount of nonspecific binding by MM oligos is dependent upon the individual nucleotide substitutions they incorporate: C→A, C→G and T→A (all yielding purine-purine mispairs) were most disruptive, whereas A→X changes had the least disruptive effect.

We also characterize a marked GC skew effect: All else equal, any substitution that raises probe GC content will exhibit higher intensity, whereas an equivalent change that lowers GC content will decrease intensity. This effect is most pronounced in mid- to low-expression regions, where nonspecific hybridization plays a larger role in total signal intensity. To prevent this skew, substitutions should be made that do not affect the GC content relative to the PM oligo (i.e., A→T, C→G, G→C, and T→A).

Multiple mismatches are largely additive in effect: both schemes (centered and staggered) conform to a near-linear decay of signal, although staggered MM are three-fold more disruptive. Each staggered MM causes > 20% intensity drop, compared with 7% per centered MM. Centered MM also display slight cooperativity, likely owing to their adjacent positioning.

RNA sample mixture complexity may affect MM binding by increasing the amount of nonspecific hybridization, thereby magnifying the GC skew and boosting the intensity of MM oligos at all levels. It does not, however, appear to affect the cost per incremental MM in either scheme. The increased mixture complexity in human is visible in increased intensity variance, particularly in non-coding regions.

These guidelines should prove useful in designing MM oligonucleotides for tiling array experiments.

## Methods

### Yeast and human genomic sequence

Yeast gene sequence coordinates were accessed via SGD  and human genic coordinates via UCSC genome browser [19]. For human sequence, repeat regions were masked out by selecting the UCSC repeat masking option on sequence retrieval.

### Yeast tiling and MM oligos

For *S. cerevisiae*, genomic regions were tiled as follows. A 10 kb double-stranded region centered around the YFL039C/ACT1 gene (chromosome 6, coordinates 48,796–59,195, 10,400 bp), was tiled with 36 mer oligos spaced every 1 bp, along with a full complement of 14 MMs (3 single-center, 5 staggered and 5 centered mismatch oligos, and one deletion oligo) per PM oligo, with PM synthesized in duplicate; then, six other genes each including +/- ~500 bp flanking regions (YBL092W/RPL32, chr2: 45,475–46,832 = 1357 bp; YGR155W/CYS4, chr7: 798,046–800,534 = 2488 bp; YOL040C/RPS15, chr15: 254,075-252,682 = 1395 bp; YOR312C/RPL20B, chr15: 901676-899780 = 1898 bp; YMR242C/RPL20A, chr13: 754,696-752,759 = 1939 bp; YLR229C/CDC42, chr12: 605,289-603,749 = 1542 bp), were tiled with 36 mer oligos spaced every 1 bp, coding strand only, PM included once, along with a smaller complement of four MMs (three single-center oligos and one deletion oligo) per PM oligo.

The total oligo count for the ACT1+flank region was 10,400 bp × 2 (double stranded) × 2 (PM in duplicate) = 41,600 PM oligos, plus 14 MM oligos per PM = 291,200 MM oligos, to yield 332,800 oligos total. For the six genes tiled single-stranded, the total oligo count was 1357 + 2488 + 1395 + 1989 + 1939 + 1542 = 10,710 PM oligos plus 4 MM oligos per PM = 42,840 MM oligos, to yield 53,550 oligos total. The total oligo count on the slide was thus 332,800+53,550 = 386,350. This slide was produced and assayed in triplicate.

### Human tiling and MM oligos

For human, genomic regions were tiled as follows. Double-stranded regions flanking exons of three main RefSeq genes (HBG2, TIMP3, SYN3) with masked repeats excluded in each case: HBG2 with 500 bp upstream and 500 bp downstream, 2464 bp total; TIMP3 first exon with 499 bp upstream and 500 bp downstream, 1722 bp total; TIMP3 last two exons with 978 bp upstream and 500 bp downstream, 5810 bp total; SYN3 first exon with 312 bp upstream and 300 bp downstream, 531 bp total; SYN3 second exon with 363 bp upstream and 502 bp downstream, 907 bp total. These regions were tiled with 36 mers spaced at 1 bp, with PM in duplicate and 13 MMs (3 single-center, 5 staggered and 5 centered mismatch oligos) per PM oligo were included. The total count for these regions was 11,434 bp × double stranded × PM in duplicate = 45,736 PM oligos, plus 13 × 22,868 = 297,284 MM oligos for a total count of 343,020. Then, two additional gene regions were tiled, also as 36 mers spaced at 1 bp, coding strand only, PM once and no MMs. These regions were: FLNA (entire gene region including introns, repeat regions removed + 1000 bp upstream and 1000 bp downstream = 25,108 oligos), FBXO7 (entire gene region including introns, repeat regions removed + 500 bp upstream and 500 bp downstream = 16,635 oligos. The total oligo count on the slide was thus 343,020 + 25,108 + 16,635 = 384,763 oligos. This slide was produced and assayed in triplicate.

### Normalization

The six slides (3 identical yeast slides and 3 identical human slides) were normalized by setting the 1.0 and 0.0 values to the median intensity in known highly expressed and lowly expressed regions, respectively, then assessing each additional intensity as a fractional value on this scale. In all cases, raw intensity values were converted to log (base 2) intensity values before this normalization process. The pairwise correlation coefficient matrices of each three-slide set (three slides each for yeast and human) were computed and showed very strong agreement (0.99+ for all pairs, data not shown).

For yeast, the upper limit was set with a 1 kb region selected from the main ACT1 exonic region (chr6: 54,387-53,388), and the lower limit was set with a 1 kb region selected from low-expression intergenic region near ACT1 (chr6: 49795-48796). The median intensities for both these regions were recorded for each of three slides, and the normalized intensity was obtained from raw intensity by subtracting the lower bound, and dividing by the difference of upper and lower bounds. Thus, for the three yeast slides (internal identifier numbers 46881, 47199 and 47202), the formulae were: slide 46881, (intensity-7.51)/6.65; slide 47199, (intensity-7.79)/6.62; slide 47202, (intensity-8.04)/6.58. Because the median high and low values were selected as bounds, some oligos have normalized intensity > 1 or < 0. The normalized PM intensity ranged from -0.281 (min) to 1.226 (max) with 70.2% of oligos falling in the range 0–1.

For human, the upper limit was set with a 0.5 kb region consisting of all HBG2 exonic regions (chr11: 5240320-5240201, 5240053-5239855 and 5238944-5238755) and the lower limit was set with the 1.8 kb intergenic balance of the HBG2 region (chr11: 5240820-5240346, 5240175-5240079, 5239829-5238970, 5238729-5238266). Normalized intensity was obtained as above. For the three human slides (internal identifier numbers 43854, 46247 and 46254), the formulae were: slide 43854, (intensity-8.19)/6.53; slide 46247, (intensity-7.69)/6.69; slide 46254, (intensity-7.66)/6.97. The normalized PM intensity ranged from -0.324 (min) to 1.195 (max) with 79.7% of oligos falling in the range 0–1.

### Arrays and hybridization

Arrays were designed using purpose-written Perl scripts and created by Nimblegen, and probed with yeast poly-A RNA and human placental RNA as per established methods [20,21].

### Media and growth conditions

Yeast strain BY4741 (leu2Δ0 ura3Δ0 met15Δ0 his3Δ1) was grown at 30C to mid-exponential phase (OD 600 = 1.0) in YPAD rich medium.

### RNA preparation

Total RNA was extracted from Ribopure Yeast kit (Ambion, Austin, TX) and treated for 30 min at 37°C with RNAse free DNase I (Ambion). Human placental poly (A)+ RNA pooled from 2–3 individuals was purchased from Ambion (Austin, TX).

### Preparation of labeled c-RNA targets

The labeling was performed using Eberwine procedure to amplify the starting material. RNA was converted to double stranded cDNA using Gibco BRL(Rockville, MD) superscript choice system and an oligo (dT) primer containing the T_7 _RNA polymerase promoter (Proligo LLC) [5'GGCCAGTGAATTGTAATACGACTCACTATAGGGAGGCGG(dT)24-3']. Briefly, 10 μg total RNA or 2 μg poly(A)+ RNA was incubated with 5× first strand buffer, 0.1 M DTT, 10 mM each dNTPs, 5 pmol primer for 60 minutes at 42°C. Second strand synthesis was accomplished by incubation with 40U DNA polymerase I, 2U of *Escherichia coli *RNase H, 10 mM ea dNTPs and 10U of *Escherichia coli *DNA ligase in 5× second strand buffer for 2 hours at 16°C. The double strand cDNA synthesis was terminated by incubating with 10U of T_4 _DNA polymerase for 5 minutes at 16°C. Double stranded cDNA was purified using phenol chloroform extraction and ethanol precipitated, washed with 80% ethanol and resuspended in 3.25 μL of water. Invitrotranscription (IVT) was used to produce biotin labeled cRNA from the cDNA using the Ambion (Austin, TX) MEGA script T_7 _kit. Briefly, 1 μg double stranded cDNA was incubated with 7.5 mM ATP and GTP, 5.625 mM CTP and UTP and 1.875 mM bio-11-CTP and bio-16-UTP (Enzo or Perkin Elmer) in 1× transcription buffer and 1× T_7 _enzyme mix at 37°C for 5 hours. Invitrotranscribed biotin labeled cRNA was purified on RNeasy mini columns (Qiagen) according to manufacturer's protocol. cRNA was quantified by absorbance at 260 nm.

### Microarray hybridization and washing

Before hybridization, cRNA was fragmented to an average size of 50–200 bp by incubating in 5× RNA fragmentation buffer (200 mM Tris acetate, pH8.1, 100 mM KOAc and 150 mM MgOAc) at 95°C for 35 minutes. Fragmentation was checked on an agarose gels. Microarrays were hybridized with 10–12 μg of cRNA in 55 μL in the presence of 40% formamide, 1 mM Tris, 0.1 mM EDTA, 5× SSC and 0.1% SDS for 18 hours at 42°C. Before application to the array, samples were heated to 95°C for 5 minutes, then at 45°C until ready for hybridization (Max 5–30 minutes). Hybridization was performed in a MAUI station.

After hybridization, arrays were washed in 0.2% SDS and 0.2× SSC for 2 minutes at 42°C and placed in nonstringent buffer (6X SSPE, 0.01%{V/V}Tween 20) until ready for the next wash in 0.2× SSC at room temperature for one minute. After washing arrays were stained with streptavidin-cy3 conjugate from Amersham Pharmacia for 25 minutes at room temperature followed by a quick rinse in 0.2× SSC and signals were amplified by antibody amplification mix(Antistreptavidin and goat IgG) for 25 minutes. Staining and amplification was repeated for 10 more minutes after a quick rinse in 0.2× SSC. This was followed by holding the arrays in nonstringent hold buffer until ready for wash in 0.2× SSC for one minute followed by 30 seconds wash in 0.05× SSC. The arrays were dried with air duster and were scanned on an Axon 4200B laser scanner at 5 μm resolution.

## Abbreviations

MM: mismatch; PM: perfect match.

## Authors' contributions

MSnyder, MG, and MSeringhaus conceived of the study. MSeringhaus designed the arrays and analyzed the data, with assistance from JR, TR and JJ. UN coordinated sample preparation and hybridization of the arrays. All authors read and approved the final manuscript.

## References

[B1] Royce TE, Rozowsky JS, Bertone P, Samanta M, Stolc V, Weissman S, Snyder M, Gerstein M (2005). Issues in the analysis of oligonucleotide tiling microarrays for transcript mapping. Trends Genet.

[B2] Southern E, Mir K, Shchepinov M (1999). Molecular interactions on microarrays. Nat Genet.

[B3] Binder H, Preibisch S, Kirsten T (2005). Base pair interactions and hybridization isotherms of matched and mismatched oligonucleotide probes on microarrays. Langmuir.

[B4] Naef F, Lim DA, Patil N, Magnasco M (2002). DNA hybridization to mismatched templates: a chip study. Phys Rev E Stat Nonlin Soft Matter Phys.

[B5] Huber W, von Heydebreck A, Sultmann H, Poustka A, Vingron M (2002). Variance stabilization applied to microarray data calibration and to the quantification of differential expression. Bioinformatics.

[B6] Irizarry RA, Hobbs B, Collin F, Beazer-Barclay YD, Antonellis KJ, Scherf U, Speed TP (2003). Exploration, normalization, and summaries of high density oligonucleotide array probe level data. Biostatistics.

[B7] Urakawa H, El Fantroussi S, Smidt H, Smoot JC, Tribou EH, Kelly JJ, Noble PA, Stahl DA (2003). Optimization of single-base-pair mismatch discrimination in oligonucleotide microarrays. Appl Environ Microbiol.

[B8] Lee I, Dombkowski AA, Athey BD (2004). Guidelines for incorporating non-perfectly matched oligonucleotides into target-specific hybridization probes for a DNA microarray. Nucleic Acids Res.

[B9] Levicky R, Horgan A (2005). Physicochemical perspectives on DNA microarray and biosensor technologies. Trends Biotechnol.

[B10] Hacia JG (1999). Resequencing and mutational analysis using oligonucleotide microarrays. Nat Genet.

[B11] Wu C, Carta R, Zhang L (2005). Sequence dependence of cross-hybridization on short oligo microarrays. Nucleic Acids Res.

[B12] Letowski J, Brousseau R, Masson L (2004). Designing better probes: effect of probe size, mismatch position and number on hybridization in DNA oligonucleotide microarrays. J Microbiol Methods.

[B13] Li X, He Z, Zhou J (2005). Selection of optimal oligonucleotide probes for microarrays using multiple criteria, global alignment and parameter estimation. Nucleic Acids Res.

[B14] Rouillard JM, Herbert CJ, Zuker M (2002). OligoArray: genome-scale oligonucleotide design for microarrays. Bioinformatics.

[B15] Rouillard JM, Zuker M, Gulari E (2003). OligoArray 2.0: design of oligonucleotide probes for DNA microarrays using a thermodynamic approach. Nucleic Acids Res.

[B16] Wang X, Seed B (2003). Selection of oligonucleotide probes for protein coding sequences. Bioinformatics.

[B17] Bozdech Z, Zhu J, Joachimiak MP, Cohen FE, Pulliam B, DeRisi JL (2003). Expression profiling of the schizont and trophozoite stages of Plasmodium falciparum with a long-oligonucleotide microarray. Genome Biol.

[B18] Nelson DC, Wohlbach DJ, Rodesch MJ, Stolc V, Sussman MR, Samanta MP (2007). Identification of an in vitro transcription-based artifact affecting oligonucleotide microarrays. FEBS Lett.

[B19] Karolchik D, Baertsch R, Diekhans M, Furey TS, Hinrichs A, Lu YT, Roskin KM, Schwartz M, Sugnet CW, Thomas DJ, Weber RJ, Haussler D, Kent WJ, University of California Santa Cruz (2003). The UCSC Genome Browser Database. Nucleic Acids Res.

[B20] Urban AE, Korbel JO, Selzer R, Richmond T, Hacker A, Popescu GV, Cubells JF, Green R, Emanuel BS, Gerstein MB, Weissman SM, Snyder M (2006). High-resolution mapping of DNA copy alterations in human chromosome 22 using high-density tiling oligonucleotide arrays. Proc Natl Acad Sci USA.

[B21] Emanuelsson O, Nagalakshmi U, Zheng D, Rozowsky JS, Urban AE, Du J, Lian Z, Stolc V, Weissman S, Snyder M, Gerstein MB (2007). Assessing the performance of different high-density tiling microarray strategies for mapping transcribed regions of the human genome. Genome Res.

